# Supplementary material for: TOPS-speed complex-valued convolutional accelerator for feature extraction and inference
Source: Nat Commun. 2025 Jan 2;16:292. doi: 10.1038/s41467-024-55321-8 (PMC11697240; doi:10.1038/s41467-024-55321-8)
Supplement: Supplementary file 1 — Supplementary Information [file 41467_2024_55321_MOESM1_ESM.pdf]

Supplementary Materials for

TOPS-speed complex-valued convolutional accelerator for feature extraction and inference

Yunping Bai,[1] Yifu Xu,[1] Shifan Chen,[1] Xiaotian Zhu,[2] Shuai Wang,[1] Sirui Huang,[1] Yuhang Song,[1] Yixuan Zheng,[1] Zhihui Liu,[1] Sim Tan,[3] Roberto Morandotti,[4] Sai T. Chu,[2] Brent E. Little,[5] David J. Moss,[6,*] Xingyuan Xu,[1,*] and Kun Xu[1,*]

[1]State Key Laboratory of Information Photonics and Optical Communications, Beijing University of Posts and Telecommunications, Beijing, China

[2]Department of Physics, City University of Hong Kong, Tat Chee Avenue, Hong Kong, China.

[3]School of Electronic and Information Engineering, Beihang University, Beijing 100191, China

[4]INRS-Énergie, Matériaux et Télécommunications, 1650 Boulevard Lionel-Boulet, Varennes, Québec, J3X 1S2, Canada

[5]QXP Technology Inc., Xi'an, China., Xi'an 710119, China

[6]Optical Sciences Centre, Swinburne University of Technology, Hawthorn, VIC 3122, Australia

*Corresponding author: xingyuanxu@bupt.edu.cn; dmoss@swin.edu.au; xukun@bupt.edu.cn

This file includes:

Supplementary Text
Supplementary Figure 1 to Supplementary Figure 16

Supplementary Note 1: Details of experiments

In this work the used optical frequency comb is a soliton crystal microcomb, which arises from the optical parametric oscillation in a single integrated micro-ring resonator (MRR). The specific process of generating the microcomb lines is slowly tuning the wavelength of the pump laser to approach the optimal position, until achieving the desired soliton crystal oscillation state. The used integrated MRR has a free spectral range (FSR) spacing of ~50.2 GHz and generates a soliton crystal with a ~0.4 nm spacing. The generated soliton crystal microcomb with a pump wavelength at 1570.62 nm offers over 90 channels within ~40 nm. The optical spectrum of experimentally generated optical frequency comb is given in Supplementary Figure 1(a). The generated Kerr microcomb operates in a stable soliton crystal oscillation state, and that the power of the comb remains constant over a certain period. In our previous work, we have measured the microcomb power stability over 66 hours, with the optical spectrum captured every 15 minutes. The extracted relative standard deviation was -14 dB over 66 hours, indicating that the microcomb source's stability can well support our convolution accelerator.

From the generated microcomb lines, we choose the half of microcomb lines inside the selected optical bands as the synaptic weights via picking one of the two adjacent lines, which are shown as Supplementary Figure 1(c) and Supplementary Figure1(d). As for the selected microcomb lines in Supplementary Figure 1(d), the same operated method to take the half of optical wavelengths as the real-part weights is conducted, and the other is used for mapping the imaginary parts of the complex-valued kernel, jointly achieving the mapping of the complex-valued kernel in HH-channel. The process can be illustrated in Supplementary Figure 1(d) and Figure 1(e). In parallel, the remaining half of microcomb lines in Supplementary Figure 1(b) will be utilized to map the complex-valued kernel in the HV-channel in the same manner.

As is shown in Supplementary Figure 1(f), the operation of selecting, shaping and de-multiplexing the microcomb lines for implementing mapping the real and imaginary parts of multiple spatially parallel complex-valued convolutional kernels are actually performed by a 1×4 waveshaper (Waveshaper 4000A), where different selected microcomb sub-bands for mapping one set of synaptic weights can be output at multiple output ports. As a result, the wavelength spacing between the adjacent wavelength channels inside real-part or imaginary part optical sub-bands is equal to the four times of wavelength spacing of the generated soliton crystal microcomb (see Supplementary Figure2(e) and Figure2(f)). At the same sequence position, the wavelength differences between two optical sub-bands are always the double of wavelength spacing of the microcomb. The novel method to select the microcomb lines for mapping the weights makes the available optical bandwidth inside the single wavelength channel significantly improved without sacrificing any microcomb line and computing parallelism. More importantly, the needed dispersive delay induced by the transmitted optical fibre obtains considerable reduction, thereby leading to effectively weakening effects of the power fading arising from the fibre dispersion. Specifically, in the designed photonic neuromorphic hardware, the occupied bandwidth of the transmitted processed signals in the single wavelength channel is about 28 GHz, inside which there is no power fading induced by the optical fibre dispersion.

As for the optical power of the microcomb and the optical losses encountered within the system, we use the optical input with the power of ~30.5dBm into the micro-ring to generate the microcomb in this work, and the total optical output power of the 18 comb lines used in the CVEOM scheme is ~20 dBm. The measured optical spectrum of the microcomb after 20dB

attenuation is shown in Supplementary Figure 1(a), and the microcomb generation system is shown in Supplementary Figure 1(b). We used an erbium-doped amplifiers (EDFA) in the CVEOM scheme, which have a total power consumption of ~200 mW, to compensate for the optical losses of the comb shaping and modulators so that the total optical input power of the photodetectors was at ~7 dBm, corresponding to a root-mean-square voltage of ~100 mVrms for the received electronic signal (with a responsivity of ~0.4 A/W and 50 Ω impedance).

The electrical signal-to-noise ratio (ESNR) of our experimental system was mainly subject to our external equipment rather than the CVOCA itself: the arbitrary waveform generator (Keysight 8196, Effective Number of Bits = 5.4) has an effective ESNR of 5.4×6=32.4 dB for the computing system; the oscilloscope (Lecory 830, vertical noise floor = 2.90 mVrms) has an effective ESNR of 20·log10(100/2.90) = 30.75 dB for the computing system. Due to limited experimental capabilities for accurate ESNR characterization, we used experimentally measured waveforms to roughly estimate the ESNR of the computing system as 36.57 dB (with <10 dB ESNR enhancement using averaging), close to the upper limit of the external equipment, thus indicating that the CVOCA would have a similar, if not higher, ESNR.

Supplementary Note 2: Network training

For the MINIST handwritten digit recognition task, data points in the top half of each image are assigned to the real part and points in the bottom half are assigned to the imaginary part. We note that here the MNIST task served as a benchmark to verify the performance of the CVOCA in convolving complex-valued data, and the real-to-complex conversion method [58] does not guarantee a better performance than real-valued operators. Further optimization of this method or alternative Fourier transform-based methods are necessary to reveal complex-valued operators' advantages for real-valued data. The training set consists of 60,000 images, and the testing set contains 10,000 images. The network includes a convolutional layer (2 kernels), a ReLU activation function, and a fully connected layer. The convolutional kernel size is 3×3, and the fully connected layer comprises 208 nodes. The convolution layer has a vertical stride of 3. We trained the network using Cross-Entropy loss for 20 epochs, employing the backpropagation algorithm with Stochastic Gradient Descent. The momentum was set to 0.9, the batch size to 64, and the learning rate to 0.01. The entire network performs 32,032 operations per image, with the convolutional layer accounting for 14,976 operations, which is 45% of the total operations needed. The in-silico recognition accuracy of the experimentally test 500 dataset is 92.8%. We note that, although the recognition accuracy did not show significant improvement, in contrast to a network with a single fully connected layer (10 neurons, each with 784 synaptic weights, 784×10=7840 real-valued synaptic weights in total) [s1], the parametric complexity can be significantly reduced (208×10=2080 complex-valued synaptic weights in the fully connected layer, 3×3×2=18 complex-valued synaptic weights in the convolutional layer).

For the SAR image recognition task, we shuffled the data in the S1SLC_CVDL dataset and then divided it into training, testing, and validation sets, with respective proportions of 80%, 10%, and 10%. Following the network structure outlined in [55], we sequentially stacked convolutional layers with batch normalization, and ReLU to construct the network structure shown in the Supplementary Figure 2(a). The pooling window's size is 2×2, the convolutional kernel's size is 3×3, and the five fully connected layers have 250, 200, 100, 50, and 7 nodes, respectively. Notably, in order to match the photonic complex-valued convolutional accelerator, the vertical stride of the

first convolutional layer that the input image passes through is set to 3. Thus, an image with a shape of 100×100 is transformed into a 34×100 shape when it passes through the first convolution layer.

We trained the network for 20 epochs using the backpropagation algorithm with Stochastic Gradient Descent, setting the momentum at 0.9, batch size at 256, and learning rate at 0.005. Cross-Entropy loss was employed, and we also clipped the norm of the gradients to 0.1. Supplementary Fig 2(b) depicts the training and validation loss curves over the course of neural network training. It can be observed that the losses for both the training and validation sets decrease as the number of epochs increases. The similar trajectories of the two curves indicate that the training can significantly optimize the performance of the neural network. Our model achieved an image recognition accuracy of 85.2% on the entire test set. For experimental testing, we randomly selected 500 images from the test set, with an accuracy of 85.4%.

We note that, the full neural network costs ~35.6 million operations for each testing sample, in which convolution costs ~32.9 million operations — >90% of the network's total needed operations, verifying the heavy computing power cost of convolution operations and thus the necessity to accelerate them.

Supplementary Note 3: Additional results

Supplementary Figures 3 and 4 show additional experimental results of input data $\mathbf{X_a}$ and $\mathbf{X_b}$,, respectively. Supplementary Figures 5 and 6 show experimentally yielded feature maps of handwritten digit images and Supplementary Figure 7 shows the confusion matrices. Supplementary Figures 8-9 show the tested 500 SAR images, where each input contains two 100×100 complex-valued images from HH-channel and HV-channel, respectively. Supplementary Figures 10-13 show experimentally yielded feature maps of SAR images. The presented images were normalized for clear visualization. Supplementary Figure 14 show the experimentally yielded additional shaped comb spectra of the SAR image recognition task

Supplementary Note 4: Potentials of performance scaling

Monolithic Integration. Although discrete components, other than the microcomb source, were used in the proof-of-concept demonstration, all components comprising of the CVOCA can be readily integrated. The microcomb itself is an integrated circuit that arises from a CMOS-compatible platform [51]. Integrated electro-optic interfaces, including modulators and photodetectors, readily support data bandwidths over 260 GHz [59-60, s2] and dual polarization modulation [s3]. The rest components of the CVOCA, including the optical spectral shaper, dispersive media, and de-multiplexer, have all been achieved based on integrated platforms [s4-s7, 62-64]. With all components integrated, the power consumption of the CVOCA mainly comes from the light source (can reach as low as 98 mW [s8]); other active devices, including modulators (thin film Lithium Niobate [s9]), photodetectors (InP [s10]), and phase shifters (thin film Lithium Niobate [s11] or doped SOI [s12]) in the optical spectral shaper, only need bias voltages and consume negligible power.

Signal bandwidth. We note that the potential analog bandwidth of input signal is subject to: a) the bandwidth of modulators and photodetectors, which can be readily achieved up to over 260 GHz [59-60, s2]; b) the Nyquist bandwidth, or half of the microcomb's free spectral range/spacing

(50 GHz for a 100GHz spaced comb source). While the more closely spaced wavelengths are utilized for implementing multiple parallel computing channels, the Nyquist bandwidth of input signal can keep enough through utilizing wavelength interleaving method. Specifically, all used wavelengths with more closely spaced can be divided into 9 groups in sequence, the single parallel computing channel can be designed by selecting one wavelength at the same position from every group to make up the weights of one 3×3 kernel. As such, in the scaled CVOCA, if a microcomb with a smaller FSR is used, stronger parallelization can be achieved without sacrificing Nyquist bandwidth via utilizing the proposed wavelength interleaving technique.

Scaling. Although the demonstrated CVOCA readily achieve high performances among complex-valued optical hardware accelerators, its parallelism and thus computing performance can be further boosted using photonic multiplexing methods and state-of-art techniques. Specifically, as shown in Supplementary Figure 15, the number of wavelength channels can be significantly increased by using broader bandwidths of microcombs, for example, over 200 wavelength channels (at a 100 GHz spacing) can be obtained when exploring the S, C and L bands (~20-THz). Two such MRRs with 50-GHz spacing difference in center pump wavelengths are interleaved and combined through a 2×1 optical coupler to produce a light source with 400 wavelengths (at a 50 GHz spacing), then split into four parallel channels via a 1×4 optical coupler. Each channel is further split by a 1×2 optical coupler and mapped to the real and imaginary weight components through the dual-polarization multiplexing, wherein the real and imaginary weights can be encoded onto the same wavelength sets via dual-polarization modulation. This can be achieved by splitting the microcomb (linearly polarized) in power into two paths (both with the same wavelengths), and separately shaped (according to desired weights $\mathbf{W}_R$ and $\mathbf{W}_I$) and modulated (with inputs of $\mathbf{X}$ and it's Hilbert transform j· $\mathbf{X}$, respectively), then one arm's polarization state is rotated to the other orthogonal polarization axis (i.e., from TE to TM) and combined together with the other arm (i.e., TE). Such architectures, using additional polarization rotators and combiners to support the polarization division multiplexing, can be readily achieved in integrated forms as demonstrated in [s3]. After spectral shaping and demultiplexing, 10 spatial parallel channels are generated, with each channel supporting four 3×3 complex-valued convolutional kernels. As such, four parallel channels include 40 spatial parallel channels in total, thereby supporting 40 3×3 kernels. For the single spatial parallel channel, the Nyquist bandwidth of input signal is over 100 GHz (can reach 500 GHz in theory) and enough for an input data rate of 100 GBaud. Therefore, the computing speed would be 100G×(2×4×9+2) = 7.4 Tops per kernel, and thus 7.4 Tops×4×10×4 = 1.184 Peta-OPs in the entire scaled CVOCA.

Here we also highlight possibilities of using other approaches to demonstrate complex-valued convolutions, such as using optical four-wave mixing [40], which achieved a computing speed of 0.15 TOPS (up to 111×30 G = 3.33 TOPS when taking into consideration of all involved computing regions). We note that this scheme cannot support fully functional complex-valued convolutions in its current form, and further investigations such as complex-valued data input and output are necessary.

Energy Efficiency. It is a challenge to directly reflect the ultimate potential of our scheme due to the fact that the CVOCA in this work was validated with discrete devices. Therefore, we have evaluated the energy efficiency of a fully integrated CVOCA, which has the same architecture as the scaled CVOCA. As shown in Supplementary Figure 15, the scaled scheme requires about 47 erbium-doped amplifiers based on integrated circuits, with each amplifier's power consumption

not exceeding 200 mW [61]. As such, the energy per operation of the scaled CVOCA can be roughly given as $(98 \times 2 + 47 \times 200)\mathrm{mW}/1.184\ \mathrm{POPs} = 0.008\ \mathrm{pJ/operation}$ —exceeding electronics (0.5pJ/operation [s15]). Supplementary Table.1 compares the power consumption of this work with existing state-of-the-art electronic or optical systems. Such performances will reach the same level of (if not exceed) state-of-art electronics [s13-s15], capable of serving as an efficient computing unit of an electro-optic hybrid computing hardware, which leverages the broad bandwidths of optics and the flexibility of electronics, ultimately achieving unparalleled performances for artificial intelligence applications.

Supplementary Table.1 The energy efficiency of the potential scaled CVOCA compared with state-of-the-art electronic or optical systems.

Reference	Power Consumption ($\mathbf{pJ/operation}$)
Nature (2021) [30]	0.8
Nature (2021) [22]	2.5
NVIDIA A100 [s16]	0.641
Google TPU-v4 [s17]	0.699
This work (the potential scaled CVOCA)	0.008

Precision. Further, we note that the input data's precision of analog optical computing hardware can be potentially much larger than their digital electronic counterparts. In contrast to the bit resolution of digital electronics that is determined by the architecture/memory width and scales with the electrical signal-to-noise ratio (ESNR) at one bit per 6dB of ESNR, optical computing hardware can potentially process data with much higher precision/bit-resolution, since: a) optical signals are less susceptible to electromagnetic interference compared to electronic signals. This can lead to clearer signal transmission and higher fidelity in data representation; b) optical computing typically generates less heat than electronic circuits, allowing for more efficient operation and the possibility of more complex systems without thermal throttling; c) optical systems can exploit principles of quantum mechanics, such as superposition and entanglement, to perform complex computations that traditional electronic systems cannot easily achieve. These advantages position optical computing hardware, including our CVOCA or more complicated optical computing hardware such as Ising machines [s18], as a promising candidate for directly processing real-world analog information (such as waves) without losing precisions due to the sampling process of digital electronics.

Other potentials. In parallel with accelerating classic operations widely achieved in digital electronics (such as the convolutions or matrix multiplication), optical computing systems' inherent physical natures (such as complex nonlinear dynamics) can be further explored to achieve

dramatically increased computing performances (trillions of physical parameters can be involved within computations in several nanoseconds), albeit with tradeoffs in terms of compatibility (such as network fan-in/-out, data formats, and universality) with existing digital electronics. We note that, while the demonstrated CVOCA performs linear operations (formed by multiplication and accumulation), it involves much more complicated nonlinear dynamics that can be further investigated and potentially harnessed to achieve dramatically different computing regimes. For example, the used microcombs arose from parametric oscillation inside a micro-ring resonator that has: a long photon life time (Q factor > 1.2 million), supporting data storage and accumulation; high nonlinearity that supports high-dimension nonlinear mapping and interference via four-wave mixing; tailored dispersion and mode-crossing that enables manipulation of the signal's linear transmission process and wavelength-dependent characteristics. Moreover, the generated soliton crystal state itself represents a mathematical solution of the Lugiato-Lefever equation, indicating that complicated physical systems can be used in turn to dramatically accelerate computing operations—if appropriate data mapping/encoding can be addressed.

Supplementary Discussion

Coherent architecture. We note that the complex-valued convolution can also be implemented via coherent architectures, which uses a single wavelength for each element of the complex-valued weight **W**, as illustrated in Supplementary Figure 16. The microcomb is first split into two paths, one path goes with an IQ modulator to load the complex-valued data's real and imaginary components, the other path goes through a Waveshaper such that the carriers' amplitudes and phases can be simultaneously manipulated to implement the complex-valued weight W. A coherent receiver is necessary to obtain the complex-valued convolution results.

The main features/differences of the I/Q modulation scheme, in contrasted to the incoherent approach demonstrated in this work, include: a) baseband modulation format that directly loads the complex-valued data **X**; b) coherent detection that requires an additional optical path to provide the LO (i.e. microcombs with complex-valued weights **W**).

We note that, while the coherent architecture uses a single wavelength for each element of the complex-valued weight **W**, it's subject to the nontrivial challenge — LO phase instability and noises, as commonly faced by coherent optical communications. This arose due to the fluctuations/noises of relative optical phases between the signal and LO paths, and requires either optical phase locked loops or/and post-DSP (for real-time phase retrieval and compensation) — both significantly increase the complexity and cost of the system, and cost nontrivial additional computing power for error/gradient calculation, phase retrieval and compensation etc.

The proposed incoherent approach, specifically the "synthetic wavelength" method, can address the issues brought about by the coherent architecture. Our incoherent approach constructs complex-valued weights **W** in a stable and incoherent manner, where the complex-valued data input and results output are independent from optical phases of the carriers (i.e., shaped microcombs), offering significantly enhanced stability, weight accuracy and robustness without additional phase locked loops or DSP. We also note that the scheme proposed in this work can also directly process waves with fast varying amplitudes and phases $\mathbf{X}[n] = |\mathbf{X}[n]|\cdot\cos\{\omega_c t+\varphi[n]\}$, rather than just complex-valued vectors $\mathbf{X}_R[n]$ and $\mathbf{X}_I[n]$ supported by the baseband IQ modulation format.

Convolution strides. In this work, the convolution accelerator we designed fundamentally operates on vectors; hence, for two-dimensional image processing applications, the input data must be flattened into vectors before processing. We utilize inhomogeneous strides, where the horizontal stride within the receptive field is set to 1, ensuring that all horizontal features from the original data are extracted; the vertical stride is equal to the height of the convolution kernel, reducing the overlap when reading the input data and partially achieving the function of pooling. Similar schemes can be referred to in the relevant sections of [30] and [s19].

Specifically, in the preprocessing stage, based on the heterogeneous strides of the receptive field, we first horizontally partition the original input matrix into multiple sub-matrices, each with a height equal to the height of the convolution kernel. We then flatten each sub-matrix into a vector from top to bottom and left to right and finally concatenate these vectors end-to-end to form a complete picture corresponding vector, ensuring that the movement of the receptive field corresponds precisely with the order of data reading.

We note that the two-dimensional data that needs to be processed during CNN processing is typically stored in corresponding storage devices. Thus, the required preprocessing merely involves reading the information from the two-dimensional data in a specific order without adding extra repetitive reads or increasing the complexity of the process. While the heterogeneous strides do not limit the performance of the convolution accelerator, as evidenced by the high recognition success rate of the CNN in our fully digital predictions, homogeneous strides can be achieved by adjusting how data is read during preprocessing or by increasing the number of accelerator spatial paths [30].

Supplementary References

[s1] Tsirigotis A., *et al,* Unconventional integrated photonic accelerators for high-throughput convolutional neural networks, *Intelligence Computing* **2,** 1-21 (2023).
[s2] Zhang, Y. *et al.* Systematic investigation of millimeter-wave optic modulation performance in thin-film lithium niobate. *Photonics Res.* **10**, 2380 (2022).
[s3] Xu, M. *et al.* Dual-polarization thin-film lithium niobate in-phase quadrature modulators for terabit-per-second transmission. *Optica* **9**, 61-62 (2022).
[s4] Carlos, Ríos. *et al.* In-memory computing on a photonic platform. Sci. Adv. **5**, eaau 5759(2019).
[s5] Cheng, Z. et al. On-chip photonic synapse. *Sci. Adv.* **3**,e1700160 (2017).
[s6] Zhou, W. *et al.* In-memory photonic dot-product engine with electrically programmable weight banks. *Nature Communications* **14**, 2887 (2023).
[s7] Zhou, W. *et al.* Phase change materials for energy efficient photonic memory and computing. *MRS Bull.* **47**, 1–9 (2022).
[s8] Cole, D. C., Lamb, E. S., Del'Haye, P., Diddams, S. A. & Papp, S. B. Soliton crystals in Kerr resonators. *Nature Photonics* **11**, 671 (2017).
[s9] Di Zhu, Linbo Shao, Mengjie Yu, Rebecca Cheng, et al., "Integrated photonics on thin-film lithium niobate," *Adv. Opt. Photon*. **13**, 242-352 (2021).
[s10] Beling, Andreas, and Joe C. Campbell. "InP-based high-speed photodetectors." *Journal of Lightwave Technology* **27**, 343-355 (2009).
[s11] Ying, Pan, et al. "Low-loss edge-coupling thin-film lithium niobate modulator with an efficient phase shifter." *Optics letters* **46**, 1478-1481 (2021).
[s12] Jacques, Maxime, et al. "Optimization of thermo-optic phase-shifter design and mitigation of thermal crosstalk on the SOI platform." *Optics express* **27**, 10456-10471 (2019).

[s13] Graves, A. et al. Hybrid computing using a neural network with dynamic external memory. *Nature* **538**, 471–476 (2016).
[s14] Yan, T. et al. All-optical graph representation learning using integrated diffractive photonic computing units. *Sci. Adv.* **8**, eabn7630 (2022).
[s15] https://coral.ai/docs/edgetpu/benchmarks/
[s16] https://www.nvidia.com/en-us/data-center/a100/
[s17] Jouppi, Norm, et al. "Tpu v4: An optically reconfigurable supercomputer for machine learning with hardware support for embeddings." Proceedings of the 50th Annual International Symposium on Computer Architecture (2023).
[s18] Mohseni, Naeimeh, Peter L. McMahon, and Tim Byrnes. "Ising machines as hardware solvers of combinatorial optimization problems." *Nature Reviews Physics* **4**, 363-379, (2022).
[s19] Krizhevsky A, Sutskever I, Hinton G E. Imagenet classification with deep convolutional neural networks. *Advances in neural information processing systems* **25**, 1097-1105 (2012).

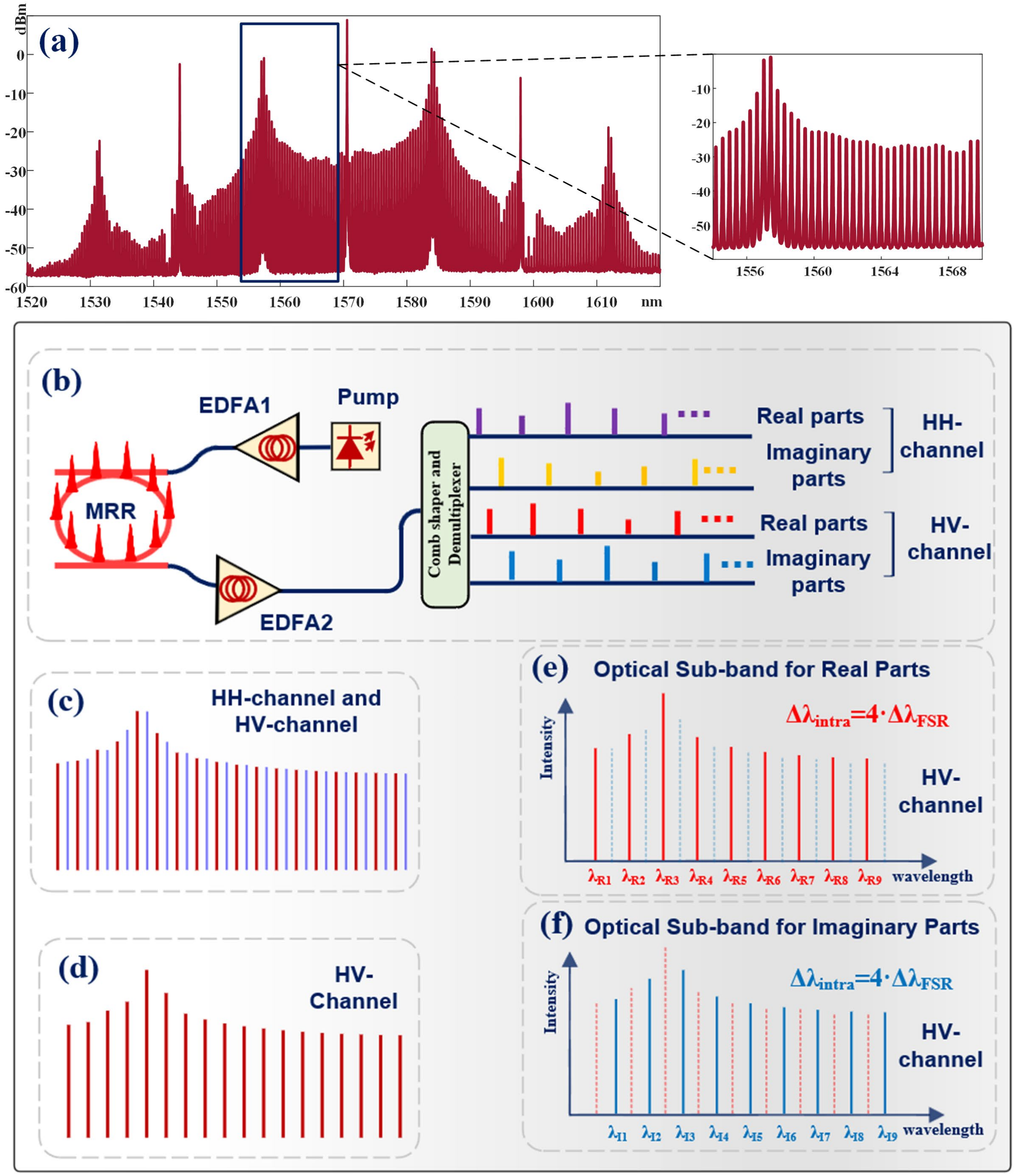

Supplementary Figure 1 | The experimentally generated soliton crystal microcomb and the selection approach of microcomb lines for mapping the real part and image part of the calculated convolutional kernel. (a) The optical spectrum of the experimentally generated soliton crystal microcomb employed in the photonic neuromorphic hardware. (b) The schematic diagram of the used approach for mapping a pair of 3×3 complex-valued kernels in the HH-channel and HV-channel. (c) The schematic diagram of selected optical band, inside that the half of microcomb lines (red lines or blue lines) is used for mapping a 3×3 complex-valued kernel, involving the real parts and image parts. (d) The schematic diagram of optical wavelength channels selected from (c), (e) and (f) the specific method to select optical sub-bands from (d) for mapping the real parts and image parts of kernel weights.

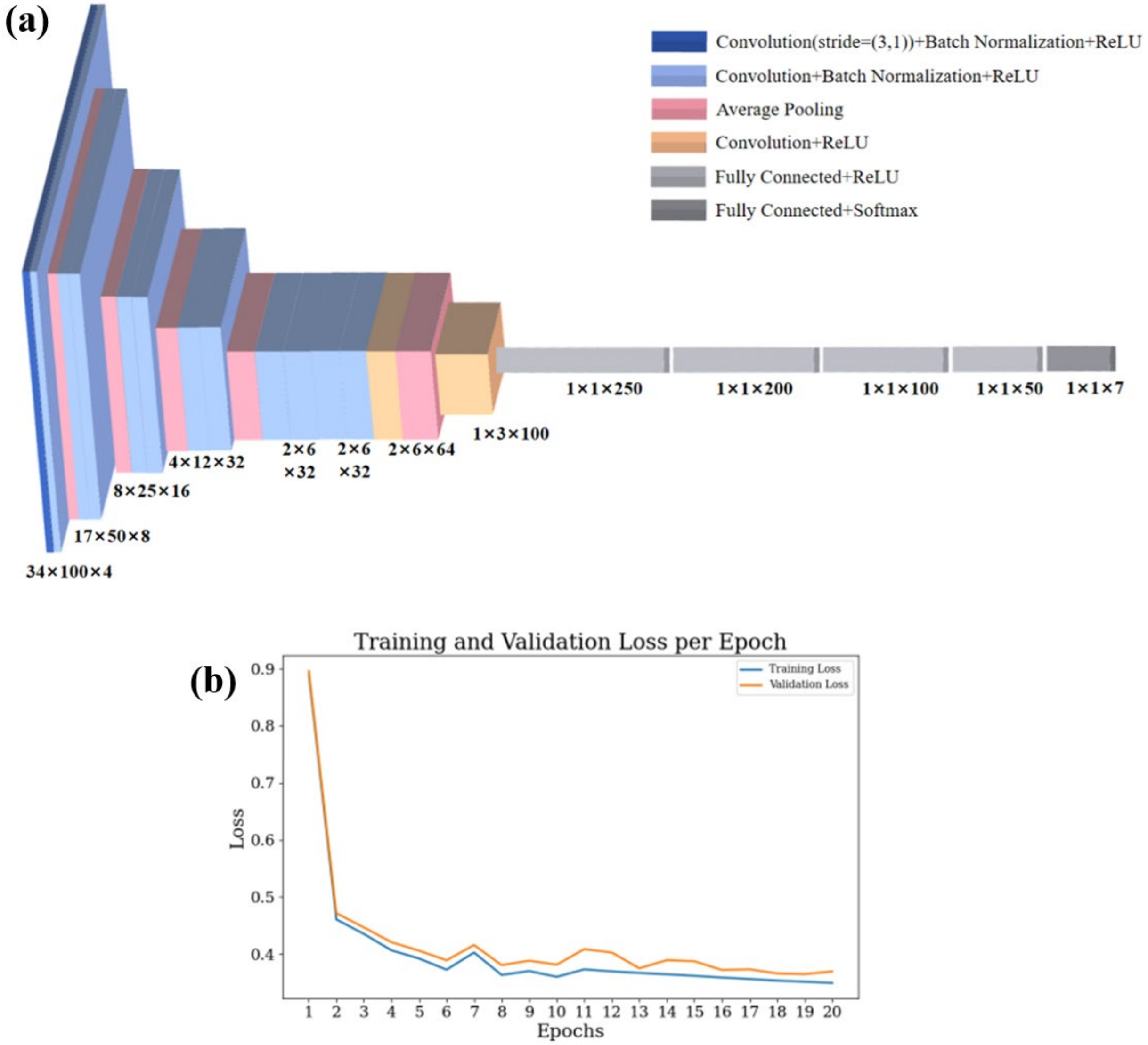

Supplementary Figure 2 | (a) the architecture of the used complex-valued convolutional neural network for SAR images classification. (b) the training and validation loss curves.

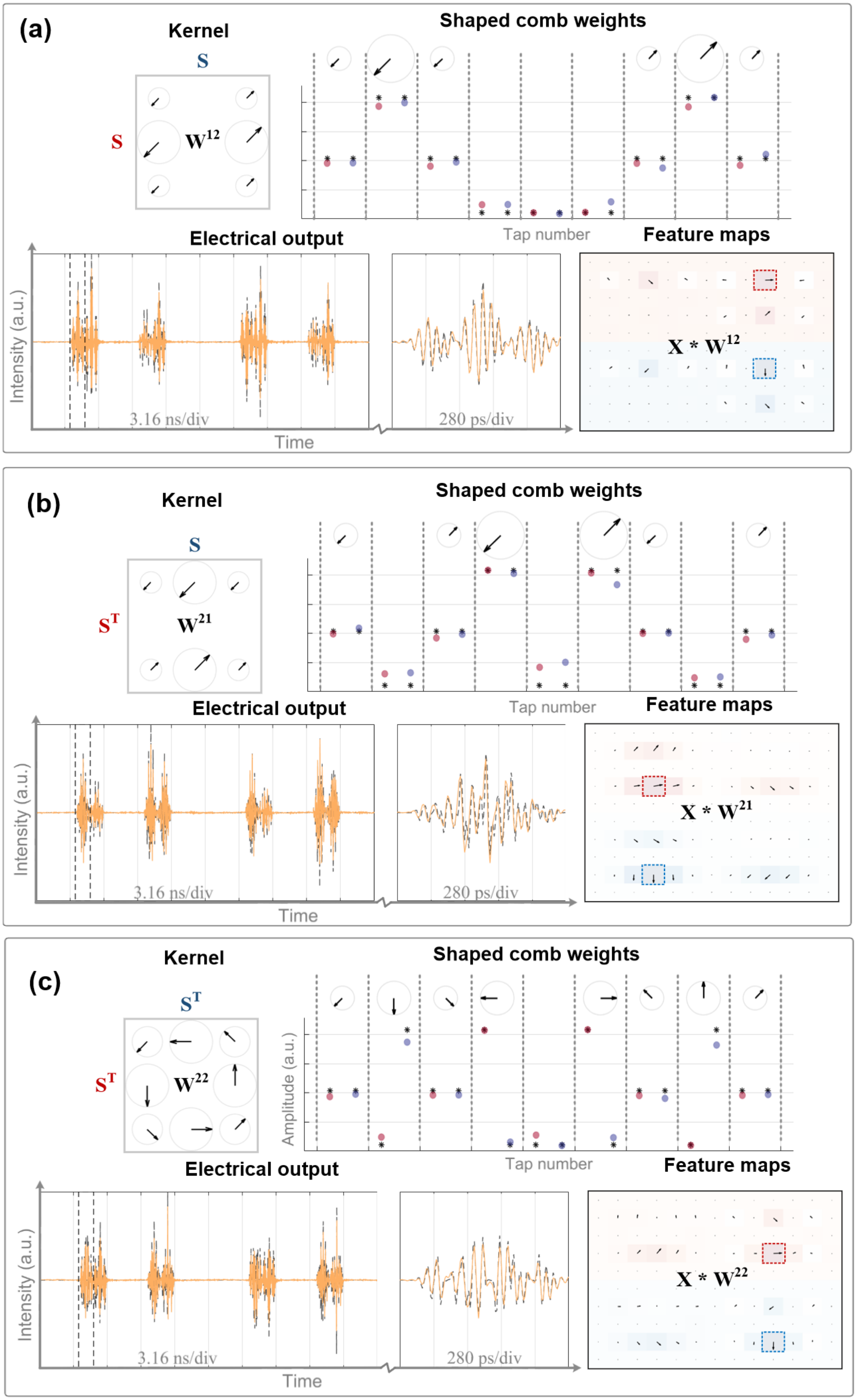

Supplementary Figure 3 | Additional experimental results of CVOCA with input data X_a.

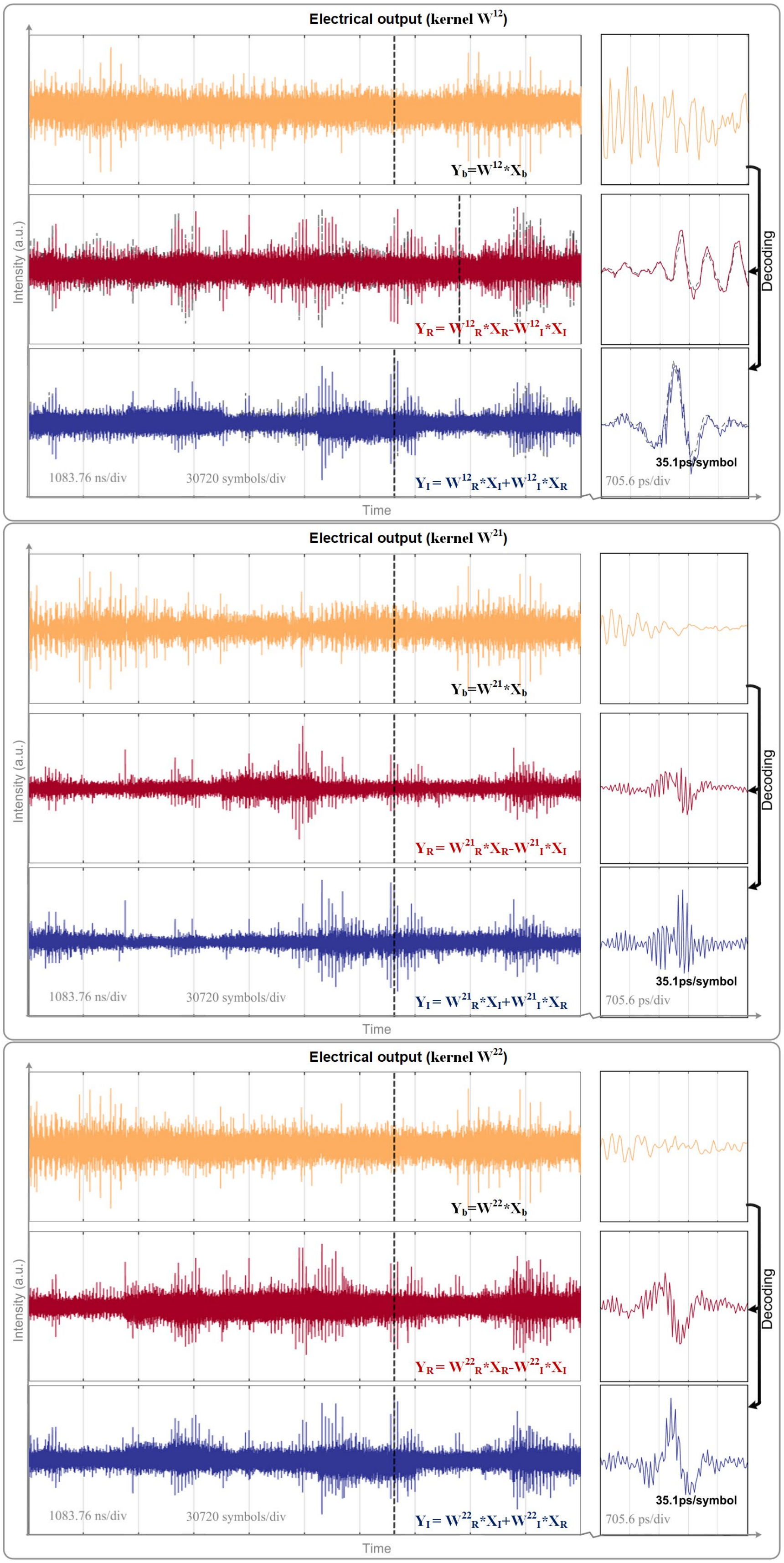

Supplementary Figure 4 | Additional experimental results of CVOCA with input data X_b.

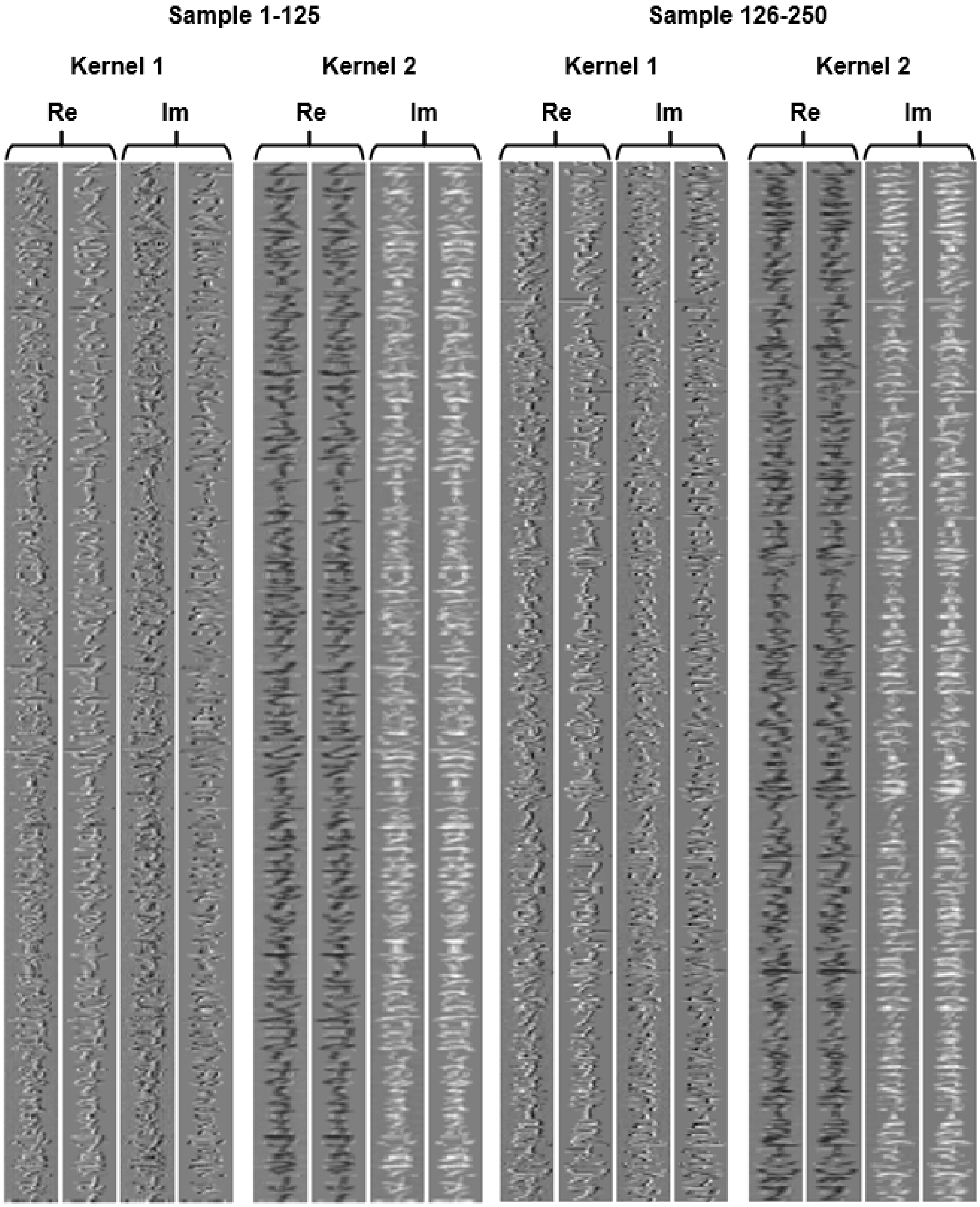

Supplementary Figure 5 | Additional experimentally yielded feature maps in handwritten digits recognition. The experimental and simulation results are given as the left and right columns of each pair.

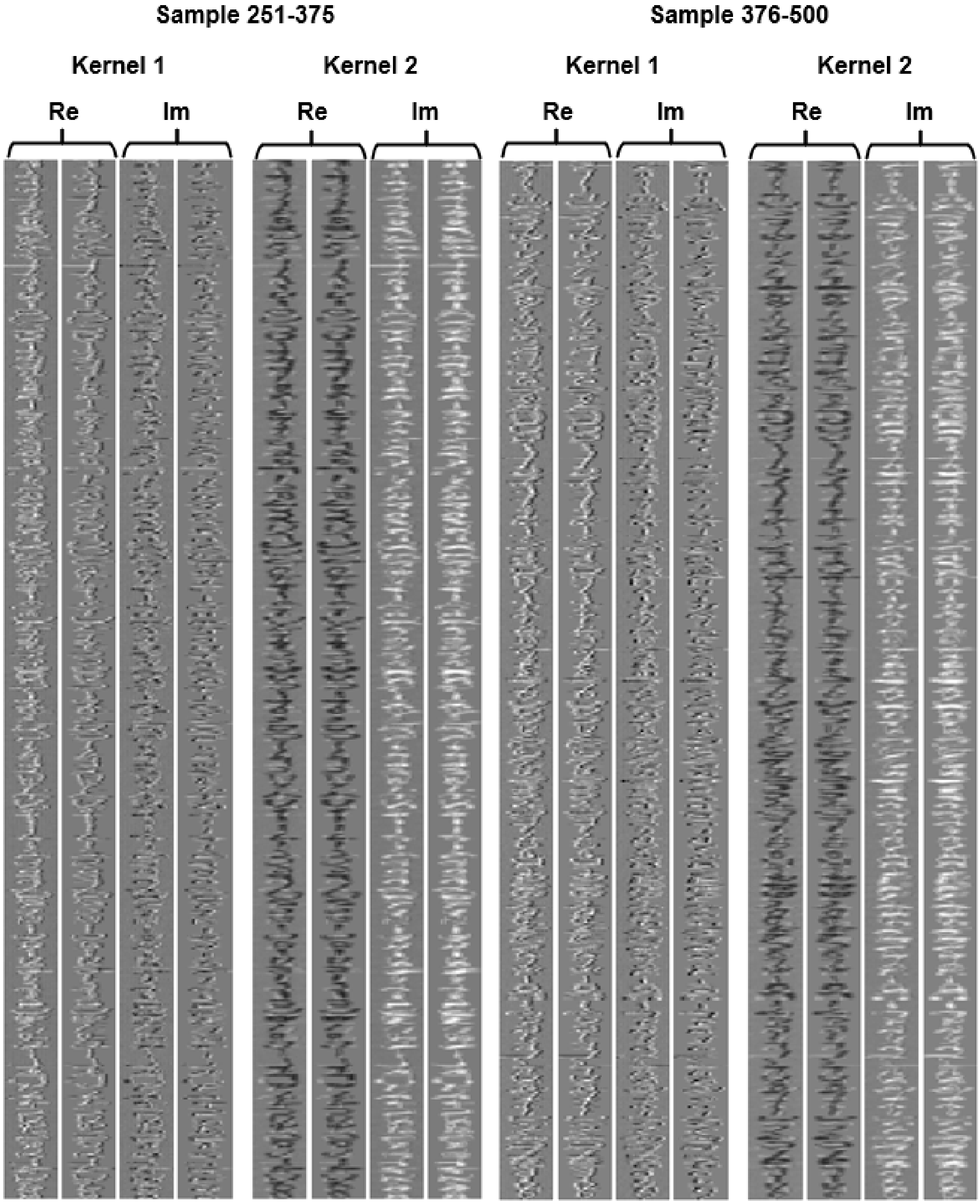

Supplementary Figure 6 | Additional experimentally yielded feature maps in handwritten digits recognition.

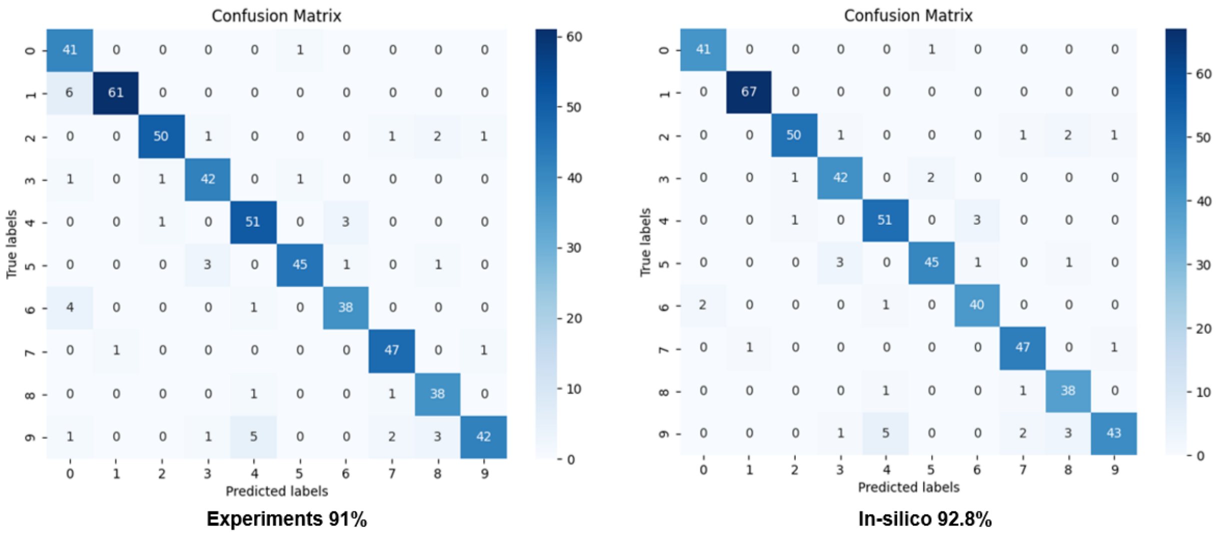

Supplementary Figure 7 | Confusion matrices of handwritten digits recognition.

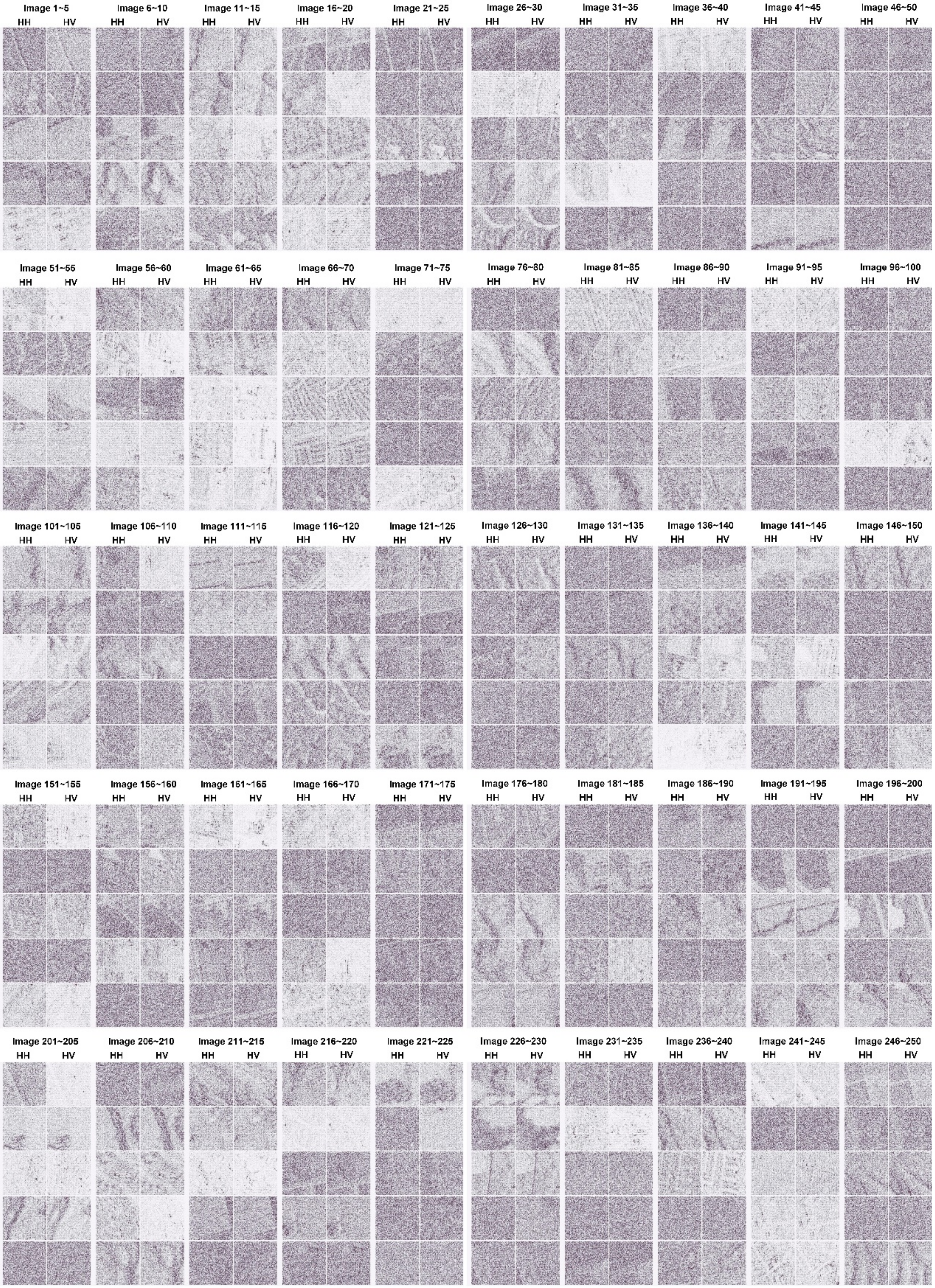

Supplementary Figure 8 | The experimentally recognized 500 SAR images that served as the input of the initial convolutional layer (1-250).

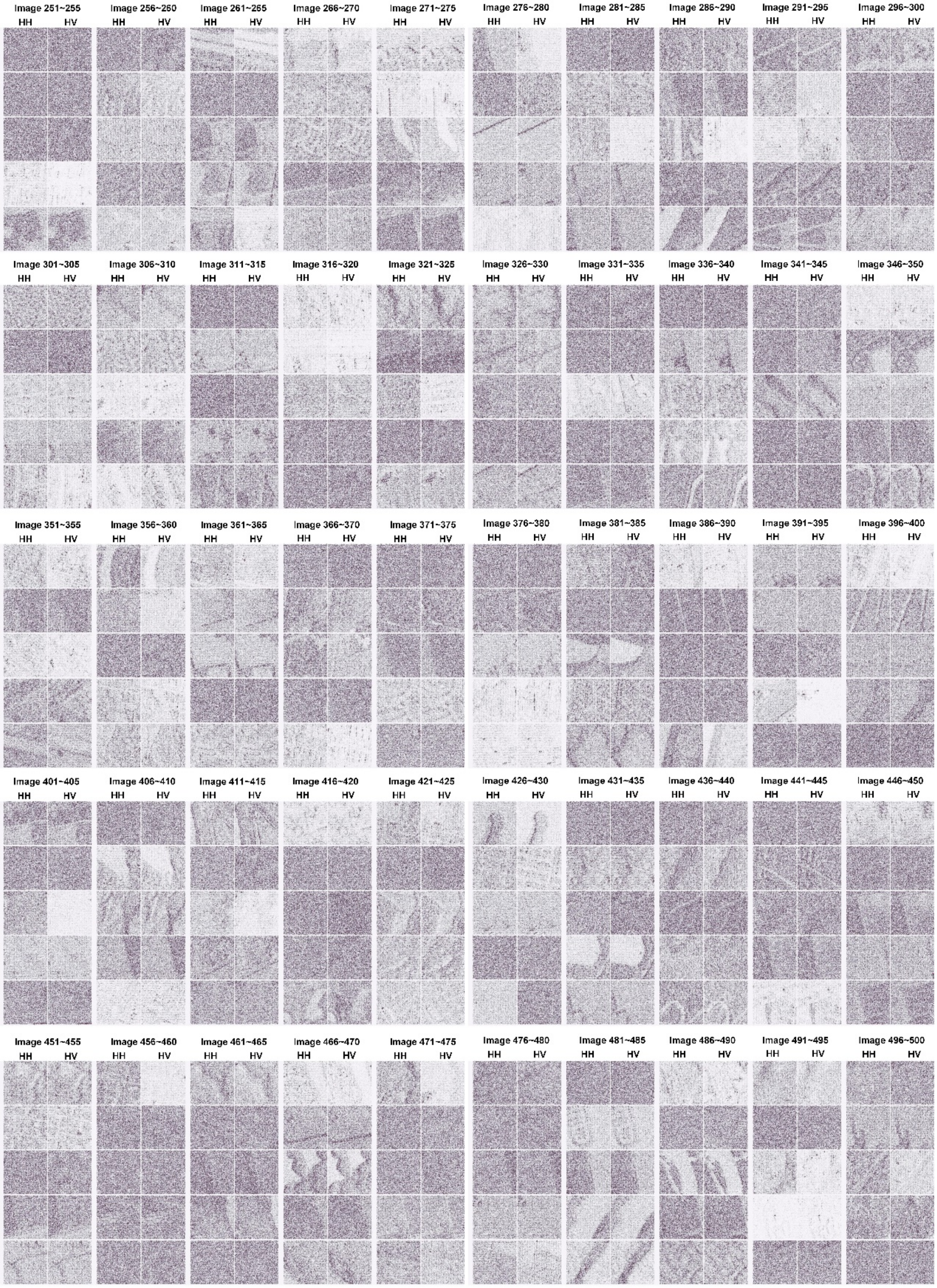

Supplementary Figure 9 | The experimentally recognized 500 SAR images that served as the input of the initial convolutional layer.(251-500).

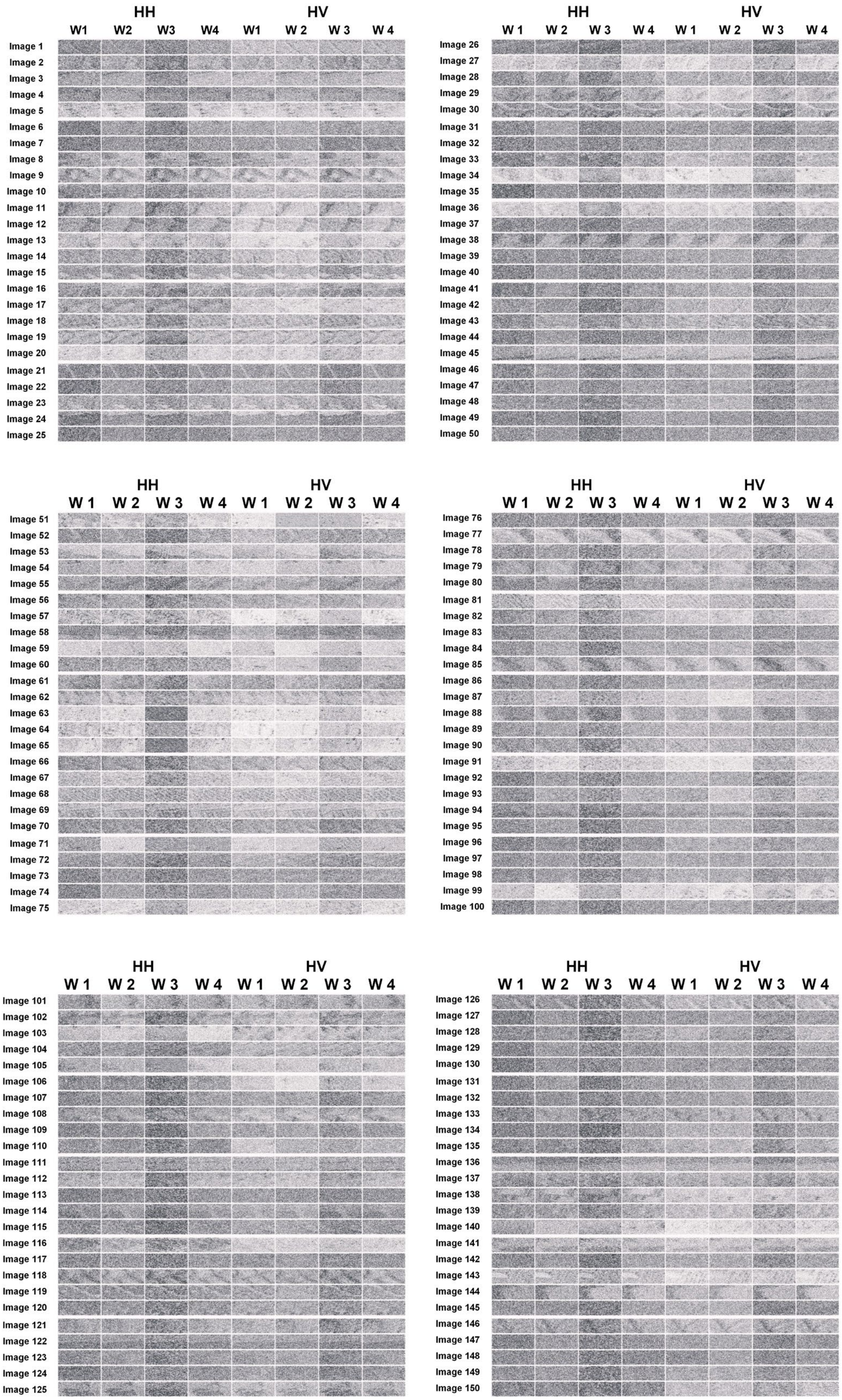

Supplementary Figure 10 | The experimentally recovered feature maps of the 500 SAR images (1-150).

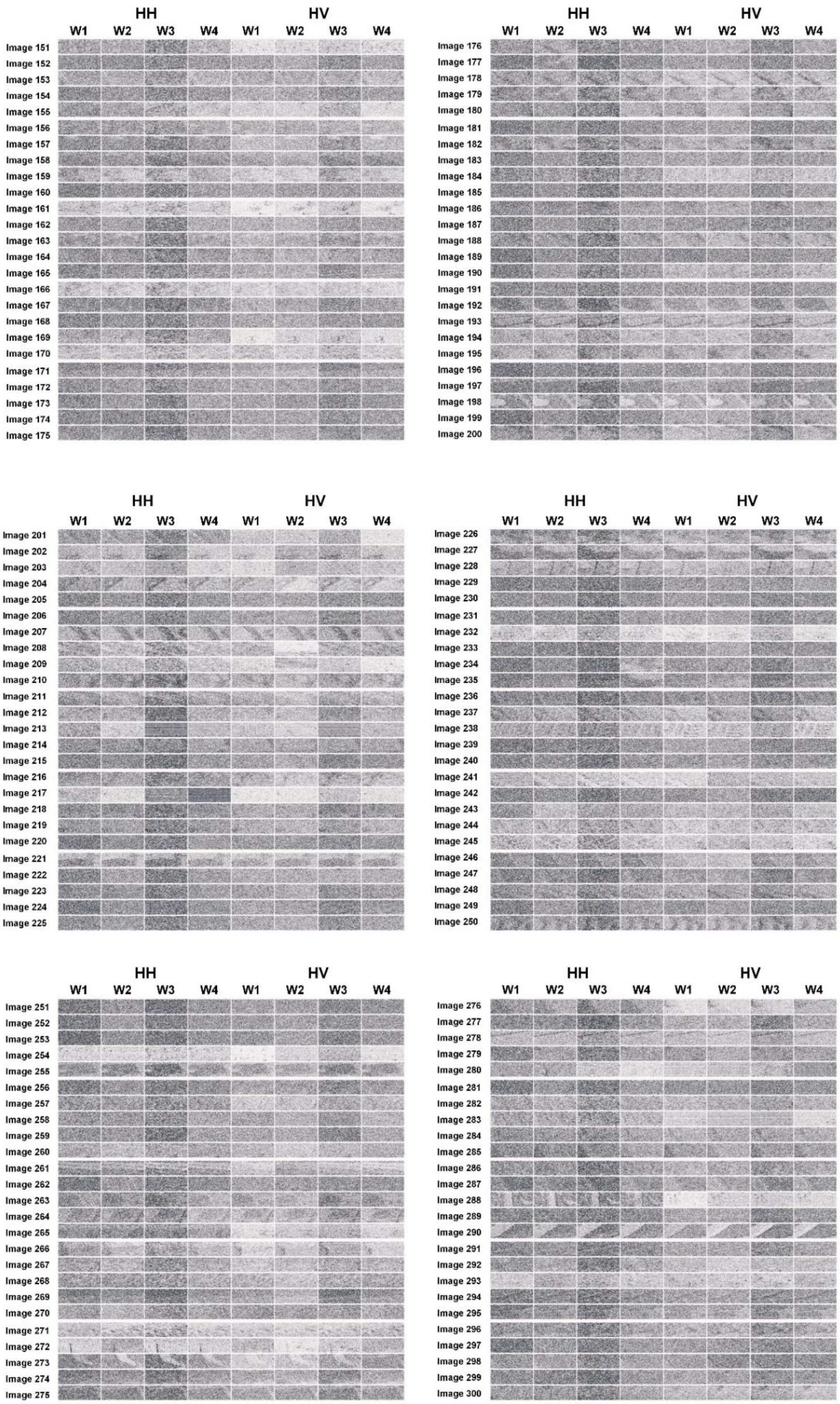

Supplementary Figure 11 | The experimentally recovered feature maps of the 500 SAR images (151-300).

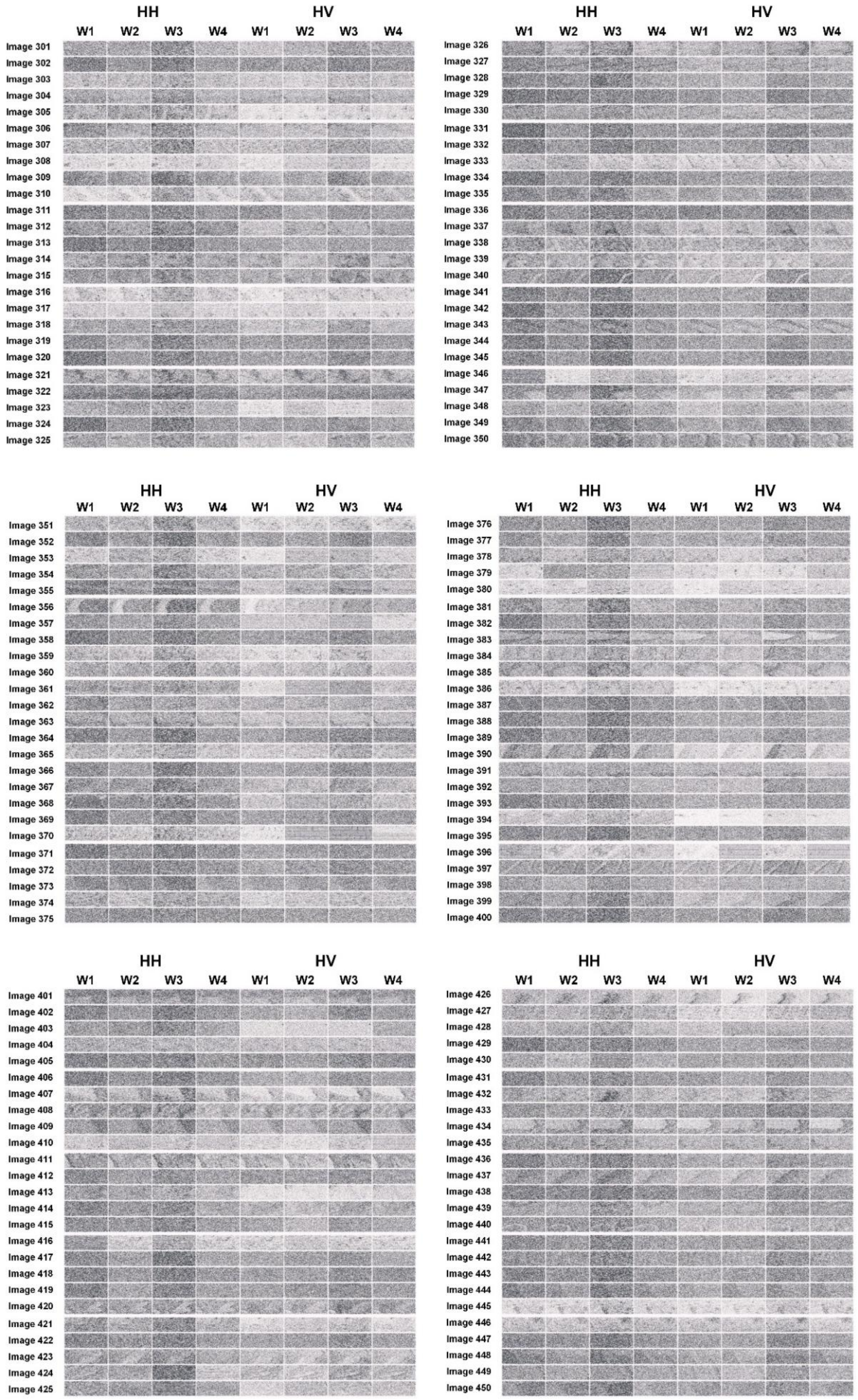

Supplementary Figure 12 | The experimentally recovered feature maps of the 500 SAR images (301-450).

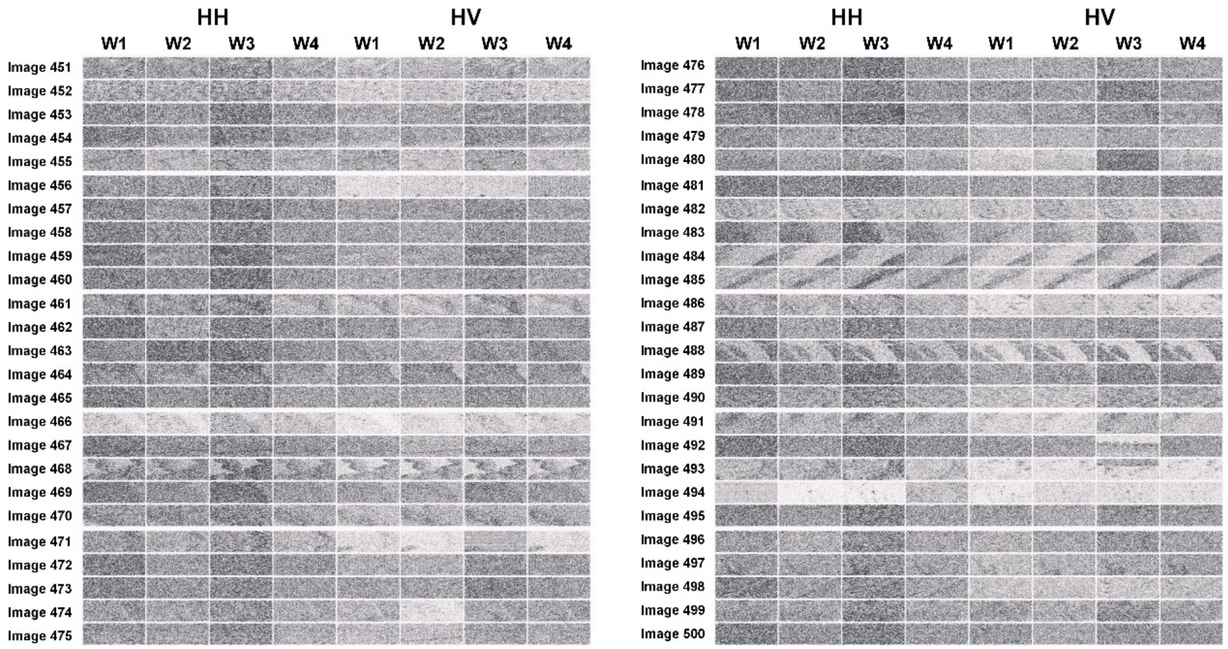

Supplementary Figure 13 | The experimentally recovered feature maps of the 500 SAR images (451-500).

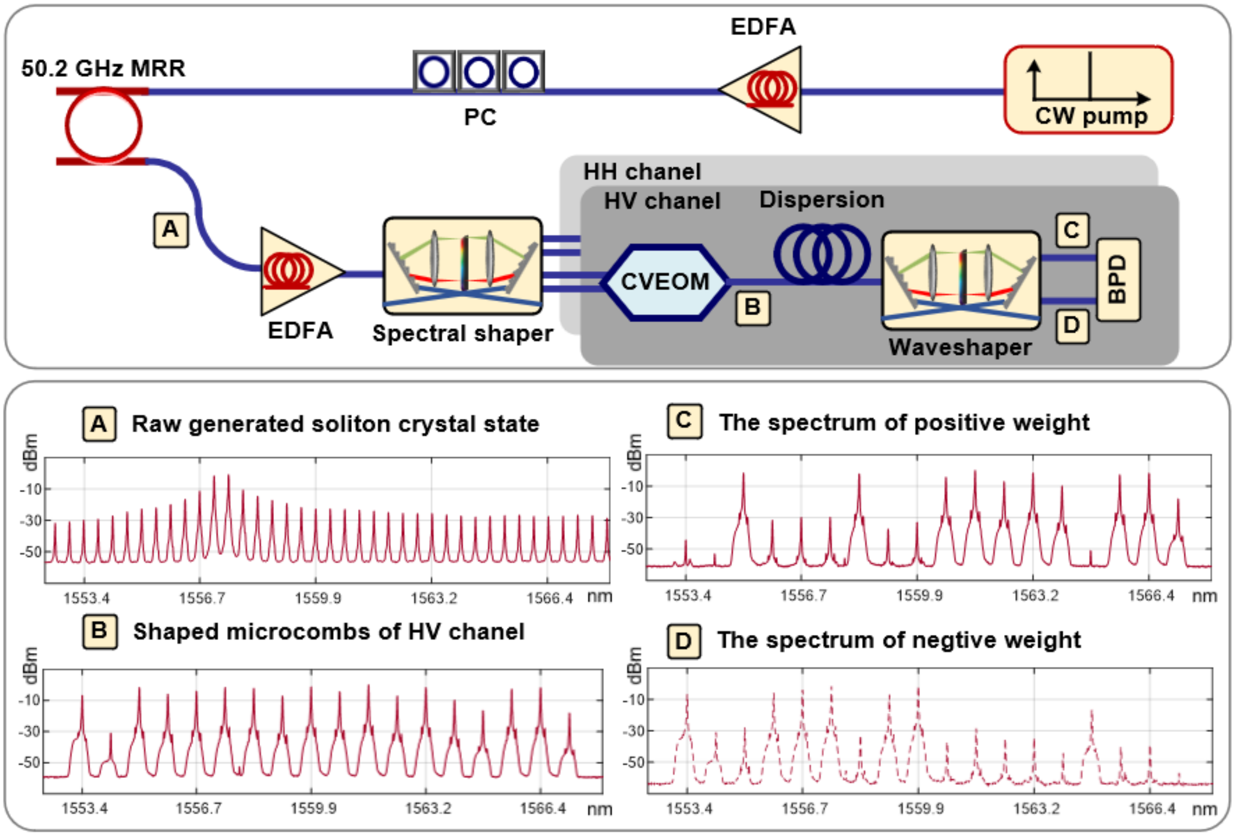

Supplementary Figure 14 | The experimentally yielded additional shaped comb spectra in the SAR image recognition task.

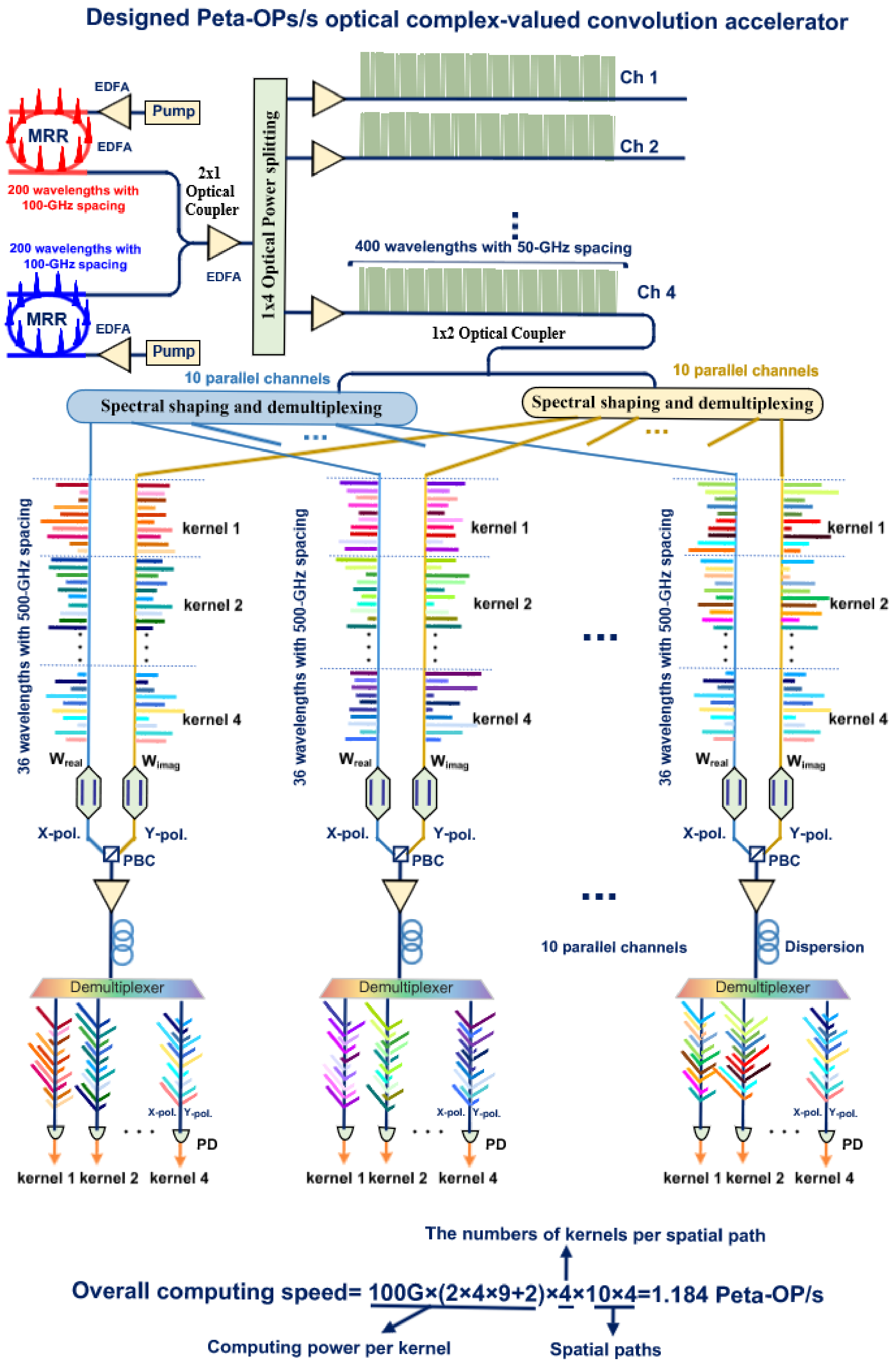

Supplementary Figure 15 | The schematic diagram of designed scaled optical complex-valued convolution accelerator by fully using the multiple freedom degrees of light.

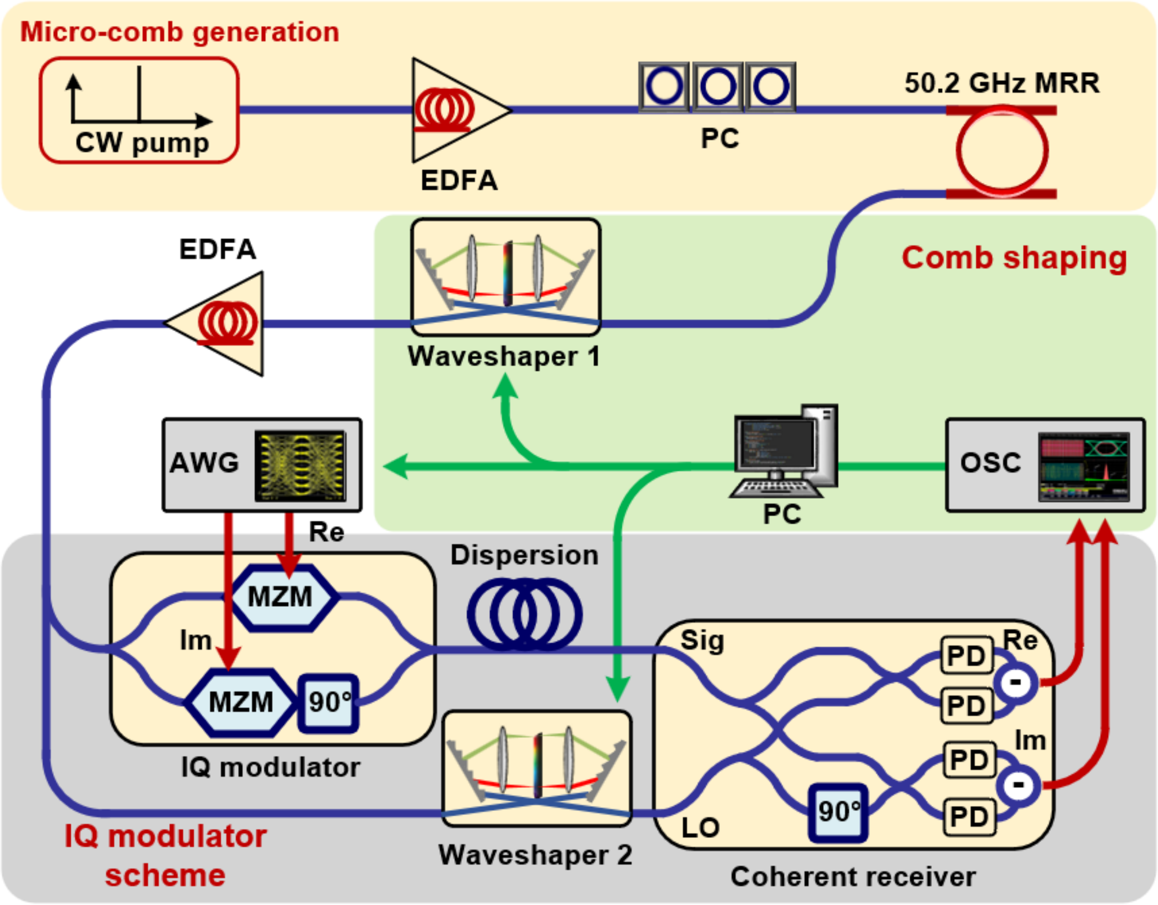

Supplementary Figure 16 | Example of coherent computing architecture for complex-valued convolution.
